# Influence of racemic higenamine on the sinus node

**DOI:** 10.3892/etm.2012.813

**Published:** 2012-11-15

**Authors:** FENGXIA YU, LINGTING KONG, SHUJUAN WANG

**Affiliations:** 1 Department of Emergency Medicine, Yantaishan Hospital, Yantai, Shandong 264001, P.R. China; 2 Electrocardiogram room, Yantaishan Hospital, Yantai, Shandong 264001, P.R. China

**Keywords:** sick sinus syndrome, racemic higenamine, sinus node recovery time, sinoatrial conduction time

## Abstract

The aim of this study was to explore the mechanism of racemic higenamine in the treatment of sick sinus syndrome (SSS). A total of 40 New Zealand rabbits were randomly divided into normal sinus node and damaged sinus node (SND) groups, and each group was randomly divided into treatment and control groups (n=10). The SND model was established by formaldehyde wet dressing of the sinus node area. The treatment groups were administered an intravenous infusion of 0.04 mg/kg racemic higenamine via the marginal ear vein within 5 min. The electrophysiological indicators of sinoatrial function, including the sinus node recovery time (SNRT), corrected sinus node recovery time (CSNRT), total sinoatrial conduction time (TSACT) and sinus cycle length (SCL), were determined before and 20 min after medication and the changes in these indicators were evaluated. The two control groups were administered 10 ml physiological saline. Following the administration of racemic higenamine, the SNRT, CSNRT, TSACT and SCL in the normal sinus node and SND groups were significantly shortened compared with those in the control groups (P<0.01). The electrophysiological influence of racemic higenamine on sinoatrial function in the SND group was significantly greater than that in the normal sinus node group (P<0.01), and its effect in the treatment of arrhythmia caused by a damaged sinus node was statistically significant (P<0.05). The main electrophysiological mechanism of racemic higenamine in the treatment of SSS was the enhancement of sinus node self-discipline and improvement of sinoatrial and atrioventricular conduction function.

## Introduction

Sick sinus syndrome (SSS) is a common disease, which is mainly caused by sinus node dysfunction. Presently, western medicine has a poor curative effect on SSS. The implantation of a permanent cardiac pacemaker may effectively relieve the clinical symptoms, but requires complex technologies, expensive costs and invasive surgery, which limits the clinical applications. Therefore, pharmacotherapy remains an important measure (1). Traditional Chinese medicine has investigated SSS from the principle of dialectical differentiation, which has numerous advantages compared with western medicine. Aconite was a commonly used traditional Chinese medicine for the treatment for SSS, and racemic higenamine has been separated from it as the effective component mixed with crudes (2). Racemic higenamine may diminish inflammation, improve blood supply to the sinus node and enhance the ventricular rate (3–5). To date, there are no studies focused on the effect of racemic higenamine on damaged sinoatrial function. In the present study, an *in vivo* damaged sinus node (SND) model was established using live animals and a direct sinoatrial electrode pacing, in order to investigate the pathogenesis of SSS, and observe the electrophysiological influence of racemic higenamine on normal and damaged sinoatrial function in rabbits, and provide an experimental basis for the traditional Chinese medicinal treatment of SSS in the clinic.

## Materials and methods

### Animal grouping

A total of 40 New Zealand rabbits were randomly divided into two groups: normal sinus node and SND groups, with 20 rabbits in each group. In the normal sinus node group (sham-operated group), a physiological saline wet dressing was applied to each of the 20 rabbits. In the SND group, 20 rabbits were used to establish damaged sinus node models by the application of a formaldehyde wet dressing. The two groups were each randomly divided into treatment and control groups. The two treatment groups were treated with 0.04 mg/kg racemic higenamine via the marginal ear vein within 5 min and after 20 min, the electrophysiological indicators of sinoatrial function were determined. These indicators included sinus node recovery time (SNRT), corrected sinus node recovery time (CSNRT), total sinoatrial conduction time (TSACT) and sinus cycle length (SCL). The two control groups were administered 10 ml physiological saline.

### Electrocardiogram recording

The electrocardiogram limb wires were inserted under the skin of limbs with electrodes (pin), and fixed with tape. Ten cardiac cycle electrocardiograms were recorded with a paper speed of 50 mm/sec.

### Establishment of the SND model

A wire twined with cotton dipped into 36% formaldehyde was placed in the border zone of the superior vena cava and right atrium for 5–10 min. An animal electrocardiogram then demonstrated sinus bradycardia, P-wave disappearance, cross-nodal rhythm and sinus arrest or tachycardia-bradycardia syndrome in succession. External application of the formaldehyde-treated dressing was stopped when tachycardia-bradycardia was observed. After stabilizing for 20 min, various electrophysiological indicators of sinoatrial function were determined. The sham operation model of normal sinoatrial function was established with physiological saline instead of formaldehyde solution. This study was carried out in accordance with the recommendations of the Guide for the Care and Use of Laboratory Animals of the National Institutes of Health. The animal use protocol was reviewed and approved by the Institutional Animal Care and Use Committee (IACUC) of Yantaishan Hospital.

### Establishment of rabbit heart model and atrial pacing algorithm

One rabbit was anesthetized with 25% urethane. A thoracotomy in the longitudinal direction was performed through the midline of the sternum and the pericardium was opened along the mediastinum to expose the right auricle as far as possible. The pacing electrode (homemade monopolar electrode) was inserted in the border zone of the root of the superior vena cava and right auricle, and another electrode was inserted into the exposed chest wall muscle to form a circuit. The other end of the electrode was connected to a DF-4A cardiac electrophysiological stimulation meter to produce cardiac pacing and determine various electrophysiological indicators of the sinus node.

### Electrophysiological determination of cardiac sinoatrial function

Ten sinus cardiac cycles were recorded and the mean was taken as the SCL. The S_1_S_1_ grading incremental pacing method was used to determine the SNRT (6). The CSNRT was calculated using the following formula: CSNRT = SNRT - basic sinus cardiac cycle. The continuous pacing method of Narula was used to measure the TSACT (7). The formula used was: TSACT = SP - PP (SP, the period from the latest pacing pulse-S to the first P-wave; PP, sinus cycle length before atrial pacing).

### Statistical analysis

Data were expressed as means ± standard deviation (SD). The means among groups were compared using one-way analysis of variance. The means of two samples were compared using a Q test. The statistics were analyzed with new drug statistical software (NDST; New Drug Statistical Treatment, Tsinghua University Publishing House, Beijing, China). The χ^2^ test was used to analyze count data.

## Results

### Establishment of models

The 40 rabbits had normal electrocardiograms prior to establishment of models. Following formaldehyde or physiological saline wet dressing of the sinus node area and stabilization for 20 min, the electrophysiological changes of the sinus node were determined, including SCL, SNRT, CSNRT and SACT (Table I).

It was observed that the sinoatrial function was significantly impaired following formaldehyde wet dressing, and SCL, SNRT, CSNRT and SACT were longer than those in the normal group (physiological saline-treated group; P<0.01), which indicated that the sinus nodes of the rabbits suffered dysfunction.

### Electrophysiology of normal sinus node

The 20 normal rabbits were randomly divided into two groups, the treatment and control groups, which were medicated with 0.04 mg/kg racemic higenamine and the control groups were administered 10 ml physiological saline via the marginal ear vein within 5 min. After 20 min, various electrophysiological indicators of sinus node function were observed to differ between the treatment and control groups (Table II).

The results demonstrate that the SNRT, CSNRT, SACT and SCL in the treatment group were significantly shorter than those in the control group, which indicated that racemic higenamine increased the normal sinus node self-discipline and accelerated the sinoatrial conduction time.

### Electrophysiology of SND rabbits

Twenty SND rabbits were randomly divided into two groups, the treatment and the control groups, after the establishment of models. The rabbits were medicated with 0.04 mg/kg racemic higenamine and the control groups were administered 10 ml physiological saline via the marginal ear vein within 5 min. After 20 min, various electrophysiological indicators of the sinus node which were compared between the treatment and the control group were observed to differ (Table III).

The results showed that SNRT, CSNRT, SACT and SCL in the treatment group were significantly shorter than those in the control group, which indicated that racemic higenamine was also able to increase the self-discipline and the sinoatrial conduction time of the damaged sinus node.

### Effects of racemic higenamine on the electrophysiology of the sinus node

Following medication of the treatment and control groups in the normal sinus node group (sham operation group), the differences in the experimental results of SNRT, CSNRT, SACT and SCL were determined, and the means and SDs were calculated. These were compared with the corresponding experimental results of the treatment and control groups in the SND group to inspect whether the affects of racemic higenamine on different sinoatrial functions of the sinus node were the same in the SND and normal sinus node groups (Table IV).

It was observed that following racemic higenamine treatment, the differences in the electrophysiological parameters between the higenamine-treated and control subgroups of the SND group were significantly larger than those of the normal sinus node group, which indicated that the affect of racemic higenamine on damaged sinus nodes was significantly greater than on normal sinus nodes.

### SND rabbit cardiac rhythm

Twenty rabbits had normal electrocardiograms prior to the establishment of models, but a variety of arrhythmia may have been present during the process of establishment. After stabilization for 20 min, the arrhythmia revealed by electrocardiograms were used as criteria to identify the therapeutic effect of higenamine on arrhythmia by comparison of the arrhythmia between treatment and control groups (using the χ^2^ test; Table V). According to the χ^2^ test, there was a statistical significance when the number of cured cases was in the range of 7–10.

## Discussion

With the continuous improvement of the standard of animal experiments, the animal model of SND with bradyarrhythmia may be established to provide an effective and scientific evaluation tool for SSS diagnosis and treatment. The acute SND model is established by formaldehyde wet dressing of the sinus node area (8,9) and is widely used at present. The establishment of a successful model is mainly determined by the statistical difference between treatment and control groups. Any of the following may be used as indications of a successful individual animal model (10): i) sinus rhythm slowed down by ∼50%; ii) atrioventricular junctional escape rhythm; iii) sinus arrest; and iv) fast-slow syndrome. SCL, SNRT, CSNRT and TSACT were significantly prolonged in the SND group compared with the normal sinus node group (all P<0.01), which showed that formaldehyde wet dressing of the sinus node area was able to cause SND and the decrease of sinus node self-discipline. The SND model was consistent with clinical SSS in electrophysiological response, stablity and reliability.

In the present study, an *in vivo* sinoatrial node damage model was established in live animals which were given direct sinoatrial electrode pacing in order to investigate the pathogenesis of SSS, and observe the electrophysiological influence of racemic higenamine on normal and damaged sinoatrial function in rabbits. The present results showed that the SCL, SNRT, CSNRT and TSACT in the racemic higenamine treatment group were significantly shortened compared with those in the control group, which indicated that racemic higenamine clearly shortened the SCL of the sinus node and reduced SNRT and TSACT. These observations are similar to the isoproterenol-like effects of B-receptor stimulants and may be associated with racemic higenamine promotion of the influx of Ca^2+^ and Na^+^ in the action potential plateau. The influx of Ca^2+^ may promote repolarizing K^+^ currents (11,12) to accelerate the speed of action potential repolarization and shorten action potential duration, which may accelerate the sinus node cells' self-discipline and sinoatrial and atrioventricular conduction. Kimura *et al*(13) investigated whether higenamine had positive chronotropic and inotropic effects on isolated rat atria. Higenamine had a cardiotonic effect on isolated heart by enhancing the myocardial contractility, increasing contraction amplitude and accelerating the sinus rhythm, and the effects on cardiac rhythm and heart rate were closely associated with the dose (14). Yu *et al*(12) observed that aconite I was clearly able to heighten Purkinje fiber self-discipline and shorten the action potential duration when perfused into the pseudo-tendon and right ventricular papillary muscle of dogs *in vitro*, but there were no significant changes to the effective refractory period. However, isoprenaline shortened the Purkinje fiber action potential duration and shortened the effective refractory period of Purkinje fiber action potential, which indicated that the mechanisms were not the same. In the clinic, isoproterenol and aconite I are both used for the treatment of various types of bradycardia and atrioventricular block. However, isoproterenol clearly shortens the effective refractory period of Purkinje fiber action potentia and, therefore, has adverse reactions such as serious arrhythmia, which may even be very serious in some cases. By contrast, aconite I is able to extend the effective refractory period of Purkinje fibers, action potential duration/effective refractory period (APD/ERP), but not shorten the effective refractory period, so that the probability of ventricular arrhythmia in clinical application is greatly decreased, indicating that aconite I may be superior to isoproterenol in the treatment of arrhythmia. This is also supported by the present study in which no ventricular arrhythmia was observed during the experimental process.

The experimental results of the SND and normal sinus node groups showed that differences in various physiological parameters of the sinus node in the SND group, including ΔSNRT, ΔCSNRT, ΔSACT and ΔSCL, were markedly larger than those in the normal sinus node group, with a significant statistical significance (all P<0.01). This indicated that racemic higenamine had a more evident effect on damaged sinus nodes than on normal sinus nodes and had a protective effect on SND. A possible mechanism is that racemic higenamine has a β-adrenergic excitatory-like effect. The mechanism may also relate to the following: i) racemic higenamine may increase coronary flow and improve the blood supply of the sinus node to improve the sinoatrial function and significantly inhibits myocardial ischemia and arrhythmia (15,16). Animal experiments have demonstrated that racemic higenamine is able to inhibit platelet aggregation, with an antithrombotic function, and improve disseminated intravascular coagulation (17,18) and myocardial ischemia-reperfusion myocardial damage. Racemic higenamine has been reported to have a marked protective effect on hypoxic-ischemic animals (19), and may reduce and relieve the range and extent of acute myocardial damage. ii) The mechanism may involve an anti-inflammatory effect. Experiments in animals have shown that racemic higenamine restrains inflammatory reaction (20,21), increases diastolic bronchial wall, increases oxygen supply and protects the cardiopulmonary function. After formaldehyde wet dressing, inflammatory exudation and protein denaturation appeared in the sinus node area, causing different degrees of damage to the tissues, blood circulation and pacemaker cell self-discipline in the sinus nodes and leading to sinus heart rate deceleration, or even arrest, ST segment shift and other ischemic changes. Following medication, racemic higenamine inhibited vascular inflammatory exudation and reduced the inflammatory changes of tissues surrounding the sinus node, which significantly inhibited the swelling of sinus node tissues. Racemic higenamine may also improve the blood supply in the sinus node area, reduce myocardial ischemia range and inhibits arrhythmia.

The experimental results of the present study confirm that racemic higenamine increases sinus node self-discipline, improves sinoatrial and atrioventricular conduction, and has a marked therapeutic effect on SSS, with few side effects. Therefore, it is of value as a clinical treatment. The study is partly limited since it is based on an acutely damaged sinus node.

## Figures and Tables

**Table I.** Electrophysiological comparison between normal sinus node and damaged sinus node (SND) groups (mean ± SD).

Groups	SNRT	CSNRT	TSACT	SCL
Normal^a^	358±67.32	100±27.33	58.12±9.71	246±23.06
SND^a^	590±89.12	190±49.63	93.2±12.9	398±52.63
Q-value	6.568	5.022	6.86	8.315
P-value	<0.01	<0.01	<0.01	<0.01

SNRT, sinus node recovery time; CSNRT, corrected sinus node recovery time; TSACT, total sinoatrial conduction time; SCL, sinus cycle length.

a 20 rabbits in total.

**Table II.** Influence of racemic higenamine on the electrophysiology of normal sinus nodes (mean ± SD).

Groups	SNRT	CSNRT	TSACT	SCL
Treatment^a^	329.6±10.99	98.2±12.11	61.1±3.81	231.4±15.03
Control^a^	362.2±24.58	112.4±15.33	70.9±3.79	251.1±1.34
Q-value	3.835	2.298	2.64	2.38
P-value	<0.01	<0.05	<0.05	<0.05

SNRT, sinus node recovery time; CSNRT, corrected sinus node recovery time; TSACT, total sinoatrial conduction time; SCL, sinus cycle length.

a 10 rabbits in total.

**Table III.** Influence of racemic higenamine on the electrophysiological parameters of SND rabbit sinus node function (mean ± SD).

Groups	SNRT	CSNRT	TSACT	SCL
Treatment^a^	511.5±56.5	147.3±34.5	81.4±16.5	364.2±29.56
Control^a^	600.0±76.1	190.7±45.5	99.8±16.2	409.3±45.53
Q-value	2.952	2.403	2.511	2.78
P-value	<0.01	<0.05	<0.05	<0.05

SND, damaged sinus node; SNRT, sinus node recovery time; CSNRT, corrected sinus node recovery time; TSACT, total sinoatrial conduction time; SCL, sinus cycle length.

a 10 rabbits in total.

**Table IV.** Comparison of the influence of racemic higenamine on the electrophysiological parameters of normal sinus node and SND group (mean ± SD).

Groups	ΔSNRT	ΔCSNRT	ΔTSACT	ΔSCL
Control^a^	32.6±25.7	14.22±14.0	9.97±2.06	19.48±15.7
SND^a^	88.5±31.3	43.4±24.4	18.4±8.41	45.1±16.1
Q-value	4.35	3.028	5.27	3.594
P-value	<0.01	<0.01	<0.01	<0.01

SND, damaged sinus node; SNRT, sinus node recovery time; CSNRT, corrected sinus node recovery time; TSACT, total sinoatrial conduction time; SCL, sinus cycle length.

a 10 rabbits in total.

**Table V.** Therapeutic effect of racemic higenamine on arrhythmia in SND rabbits.

Groups	Cured	Not cured	Total
Control	1	9	10
Treatment	9	1	10
Total	10	10	20

SND, damaged sinus node.
